# Isoprostanes-Biomarkers of Lipid Peroxidation: Their Utility in Evaluating Oxidative Stress and Analysis

**DOI:** 10.3390/ijms11114631

**Published:** 2010-11-17

**Authors:** Monika Janicka, Agata Kot-Wasik, Jacek Kot, Jacek Namieśnik

**Affiliations:** 1 Department of Analytical Chemistry, Chemical Faculty, Gdańsk University of Technology, Narutowicza 11/12, 80-233 Gdańsk, Poland; E-Mails: agata@chem.pg.gda.pl (A.K.-W.); jacek.namiesnik@pg.gda.pl (J.N.); 2 Department of Hyperbaric Medicine and Sea Rescue, Interdepartmental Institute of Maritime and Tropical Medicine, Medical University of Gdańsk, Powstania Styczniowego 9B, 81-519 Gdynia, Poland; E-Mail: jkot@acmmit.gdynia.pl

**Keywords:** isoprostanes, exhaled breath condensate, biomarkers

## Abstract

Isoprostanes (IsoPs) are key biomarkers for investigating the role of free radical generation in the pathogenesis of human disorders. To solve IsoPs-related problems with regard to isoprostanes, analytical tools are required. This paper reviews the problems and trends in this field focusing on the methodology for assaying biomarkers in exhaled breath condensate (EBC) samples. A large amount of work has been done in the qualitative and quantitative analysis of IsoPs, but a standardized method has yet to emerge. The methodologies described differ, either in the sample preparation steps or in the detection techniques, or both. Requiring a number of chromatographic steps, the relevant extraction and purification procedures are often critical and time-consuming, and they lead to a substantial loss of target compounds. Recent data show that EBC is a promising non-invasive tool for the evaluation of different diseases. Two main analytical approaches have been adopted for IsoPs measurement: immunological methods and mass spectrometry. The methodologies for the extraction, purification and analysis of IsoPs in EBC samples are presented.

## 1. Introduction

**Figure 1.**
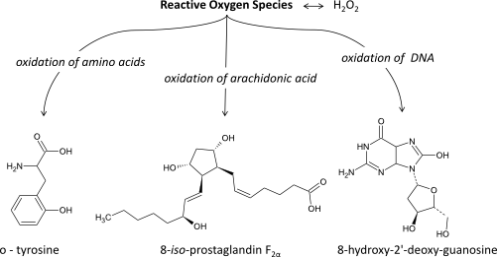
Main products of Reactive Oxygen Species activity.

In recent years, oxidative stress has been implicated in various pathological situations. The difficulty is to choose a convenient marker for evaluating its importance *in vivo*, because of analytical problems of specificity and sensitivity. The oxidized lipids formed during lipid peroxidation, illustrate these problems (Figure 1). Among these markers are ‘primary’ products such as hydroperoxides, or ‘secondary’ products such as malondialdehyde (MDA), 4-hydroxynonenal (4-HNE) and isoprostanes (IsoPs). They are all measurable in biological fluids, but analytical procedures used are sometimes complex and require sample preparation involving extraction and purification steps. IsoPs are certainly the most specific markers of lipid peroxidation but they are also most difficult to measure. Several comprehensive reviews providing information on the biochemistry of IsoPs and their utilization as a marker of oxidative stress have been published in the last 10 years [1–5]. Numerous assays have recently been developed: some, like gas or liquid chromatography coupled with mass spectrometry, are the ‘gold standard’ methods, enabling different IsoPs to be measured but they required special apparatus. Others, like immunoassay methods, measure one IsoP—they are simpler to perform and are accessible to a greater number of laboratories but they are still lacking in specificity.

**Figure 2.**
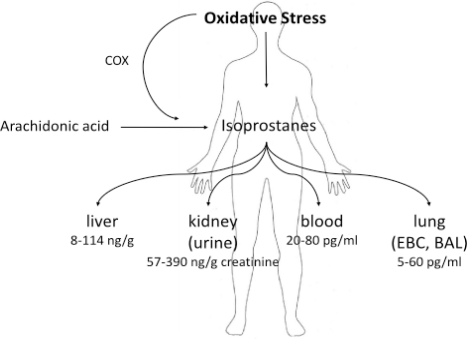
Isoprostane levels in different specimen.

The materials used to measure isoprostanes and their metabolites are urine [6–9], blood plasma [8,10–11], cerebrospinal fluid [12], exhaled breath condensate [13–17], bronchopulmonary lavage (BAL) [18], amniotic fluid [19], meconium and tissues [20]. Several systemic diseases, such as atherosclerosis, hypertension and others, were described to increase serum and urine 8-isoPGF_2α_ level [21]. The IsoP levels found in different specimen are summarized in Figure 2.

The discovery of IsoPs has important implications for medicine:

- It has now been established that measurement of IsoPs is the most reliable approach to the assessment of oxidative stress status *in vivo*, providing an important tool for exploring the role of oxidative stress in the pathogenesis of human disease;
- products of the IsoPs pathway have been found to exert potent biological actions and therefore may be pathophysiological mediators of disease.

In the mid-1990s a new, non-invasive sample collection technique for investigating the lungs was brought to the attention of researchers: exhaled breath condensate (EBC). It shows the extent of interest that more than 80 original articles have been published on this subject in the last five years. Many substances are found in expired breath that are detectable in the liquid that can be obtained by cooling (*i.e.*, condensing) it. The advantages of this method are that it is non-invasive, convenient, and can be carried out on mechanically ventilated patients as well as on children

There are many compounds that are determined in EBC: volatile compounds (VOCs) [22], non-volatile compounds (very low molecular weight compounds, low molecular weight compounds, polypeptides, proteins, nucleic acids), non-volatile compounds derived from volatile compounds, and miscellaneous others (lipid mediators, inorganic molecules, organic molecules, redox relevant molecules, pH relevant molecules, cytokines, chemokines). It is important to note that some clearly non-volatile compounds found in EBC may be derivatives of volatiles. For example, nitrate (NO_3_^−^) and nitrite (NO_2_^−^), ionized and therefore not volatile, may arise in EBC in part from a reaction of volatile nitric oxide gas (NO) after reaction with oxygen [23]. Volatiles such as acetic acid, formic acid and ammonia are found in much higher concentrations in EBC than non-volatile constituents, and are thus much easier to measure.

The non-volatile constituents of EBC make up a broad category, containing molecules as small as sodium ions (Na^+^) and as large as immunoglobulins. There are numerous publications in the literature presenting an individual compound that has been found in EBC (often relying on one assay) with levels depending upon the disease state, and comments that the biomarker may be valuable in managing the disease of interest. The most widely studied substance is hydrogen peroxide, which is a marker of oxidative stress: Its level in EBC is elevated in numerous inflammatory diseases.

**Figure 3.**
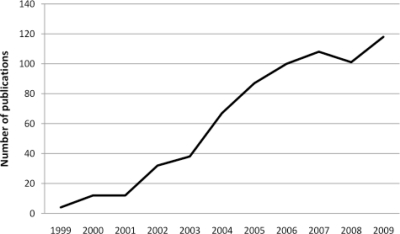
Number of publications on EBC over the past 10 years.

A search of the Information Sciences Institute (ISI Web of Knowledge) revealed some 855 scientific publications concerning EBC (to June 14, 2010) in the peer-reviewed literature. However one should expect that even 100 more publications are identified in the non-peer-reviewed literature. Although one of the first papers was published back in 1980, the number of publications exhibiting increased concern regarding EBC has risen fairly steadily over the past 10 years (see Figure 3).

The number of articles on EBC analysis has increased tenfold in the last 10 years.

## 2. Isoprostanes as Biomarkers of Diseases

The measurement of isoprostanes (IsoPs) in biological fluids and/or tissue specimens has important clinical implications. Oxidative stress is the hallmark of various chronic inflammatory lung diseases. Increased concentrations of reactive oxygen species (ROS) in the lungs of such patients are reflected by elevated concentrations of oxidative stress markers in the breath, airways, lung tissue and blood. Traditionally, the measurement of these biomarkers has involved invasive procedures to procure the samples or to examine the affected compartments, to the patient's discomfort. As a consequence, there is a need for less or non-invasive approaches to measure oxidative stress. The collection of exhaled breath condensate (EBC) has recently emerged as a non-invasive sampling method for the real-time analysis and evaluation of oxidative stress biomarkers in the lower respiratory tract airways. Biomarkers of oxidative stress such as H_2_O_2_, IsoPs, malondialdehyde, 4-hydroxy-2-nonenal, antioxidants, glutathione and nitrosative stress markers such as nitrate/nitrite and nitrosated species have all been successfully measured in EBC.

Measurements of IsoPs in EBC have several advantages over other markers of oxidative stress: They are chemically stable, are formed *in vivo*, are specific to lipid peroxidation, and are used to define the clinical pharmacology of antioxidants [24].

**Figure 4.**
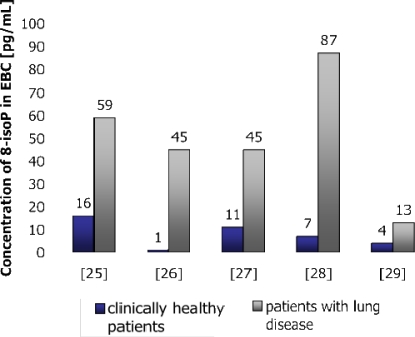
Concentration of 8-isoPGF_2α_ in EBC in healthy and non-healthy subjects [25–29].

8-IsoP concentration was found to be elevated in asthma [30–33], COPD [34], interstitial lung disease [35], cystic fibrosis (CF) [36], acute respiratory distress syndrome (ARDS) [28], pulmonary sarcoidosis [37], obstructive sleep apnea [38], and also in healthy subjects after ozone-inhalation [39]. However, in asthma, the observed increase in the EBC 8-isoPGF_2α_ level was related to the severity of the disease, and the relative resistance of 8-isoPGF_2α_ to steroids has been reported in children with asthma exacerbation [31–33]. 8-isoPGF_2α_ levels were also found to be elevated during COPD exacerbation, but fell after treatment [34]. Differencies between IsoPs concentrations in healthy and non-healthy subjects are presented in Figure 4.

Exhaled breath condensate is obtained from both adult and child patients suffering from various pulmonary diseases such as asthma, cystic fibrosis, chronic obstructive pulmonary disease, and interstitial lung diseases.

**Figure 5.**
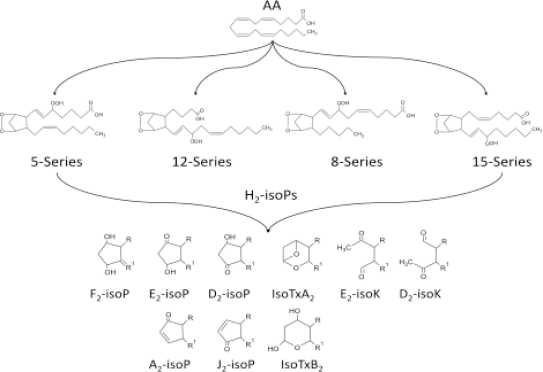
Products of free radical-induced peroxidation of arachidonic acid.

Several *in vitro* markers of oxidative stress are available, but most are of limited value *in vivo* because they lack sensitivity and/or specificity or require invasive methods. IsoPs are prostaglandin (PG)-like substances that are produced *in vivo* independently of cyclooxygenase (COX) enzymes, primarily by free radical-induced peroxidation of arachidonic acid (Figure 5).

An important aspect of the discovery of IsoPs is that their measurement has emerged as one of the most reliable approaches to assess oxidative stress status *in vivo*, providing an important tool to explore the role of oxidative stress in the pathogenesis of human disease.

Isoprostanes F_2α_ are a group of 64 compounds isomeric in structure to cyclooxygenase-derived PGF_2α_. Other products of the IsoPs pathway are also formed *in vivo* by rearrangement of labile PGH2-like IsoP intermediates.

The discovery of IsoPs has important implications for medicine: First, it has now been established that measurement of IsoPs is the most reliable approach to assess oxidative stress status *in vivo*, providing an important tool to explore the role of oxidative stress in the pathogenesis of human disease. In addition, products of the IsoP pathway have been found to exert potent biological actions and may therefore be pathophysiological mediators of disease [40].

### 2.1. Applications of Measurement of 8-IsoPGF_2α_ in Lung Diseases

Although measurement of 8-isoPGF_2α_ in EBC is a useful noninvasive approach for exploring the role of oxidative stress in lung diseases (this technique might provide important insights into the understanding of the clinical pharmacology of antioxidants and might be useful for monitoring the effects of pharmacological therapy [41]), little is known about whether the concentration of oxidative stress markers is also affected by some systemic diseases (atherosclerosis, diabetes, *etc.*). Another unanswered question is the locus of the origin of these markers in the respiratory tract. No positive correlation between blood and EBC concentrations was seen in the studied markers. No significant difference was seen in the level of the markers at different breathing frequencies.

Markers of oxidative stress in the EBC seem to be independent of blood levels, which confirms their importance for lung disorders. The results are compatible with the hypothesis that the source of increased EBC markers is the lung parenchyma.

#### 2.1.1. Allergy

Allergic rhinitis is a common disease that is reported to affect 10–40% of the global population and its prevalence is increasing in both adults and children. There are significant differences between documented studies on 8-IsoP measurement in patients with this disease. One study shows no differences between the study groups in the levels of 8-IsoP in EBC of controls and allergic rhinitis patients [42], whereas in another 8-IsoP was significantly increased in a group of patients with allergic rhinitis (mean 43.6 pg/mL) than in healthy subjects (mean 18.6 pg/mL) [43].

#### 2.1.2. Asbestos Exposure

Asbestos exposure leads to asbestosis and to the development of pleural plaques; it also increases the risk of mesothelioma and lung cancer. Asbestosis and pleural plaques exhibit unpredictable but progressive development, and there are no markers routinely available for assessing their prognosis. Asbestos exposure induces the generation of reactive oxygen species, and 8-IsoP is involved in experimental asbestos related lung toxicity. This oxidative stress marker was measured in EBC in former asbestos workers. The mean level of 8-IsoP in asbestos-exposed subjects reached the level of 69.5 ± 6.6 pg/mL and was higher compared with the control group, where the concentration was 47.0 ± 7.8 pg/mL. The results presented support the hypothesis that oxidative stress due to asbestos is the main cause of increased 8-IsoP in EBC. Measurement of 8-IsoP in EBC is thus a promising non-invasive means for assessing the activity of asbestos-induced diseases [16]. Another study, with the aim of assessing oxidative stress and lung inflammation *in vivo* by measuring 8-IsoP in exhaled breath condensate from humans with asbestosis, showed that in asbestos-related disorders, markers of inflammation and oxidative stress were significantly elevated in subjects with asbestosis compared with healthy individuals but not in pleural diseases [44].

#### 2.1.3. Asthma

8-IsoPGF_2α_ is detectable in the exhaled breath condensates of children with asthma (asthmatic airways are characterized by enhanced oxidative stress, which can therefore be studied by measuring 8-IsoP) and may be used as a non-invasive measurement of oxidative stress in childhood asthma. Measurements of 8-IsoP concentrations in EBC and urine of children with problematic and well-controlled asthma showed that 8-IsoP in EBC was significantly elevated in children with problematic asthma [33]: 8-IsoP levels measured in urine, however, did not correlate with those measured in EBC [45]. The persistence of high levels of 8-IsoP despite the inhalation of steroids suggests that other treatments such as anti-oxidants might be beneficial.

Lung oxidative stress is increased in children who are in a stable condition with asthma. 8-IsoP was detectable in the EBC of healthy children (mean, 34.2 ± 4.5 pg/mL), but elevated levels were found in both steroid-naive asthmatic children (mean, 56.4 ± 7.7 pg/mL) and steroid-treated asthmatic children (mean, 47.2 ± 2.3 pg/mL) [31]. However, EBC analysis cannot provide any information on the cellular origin of mediators. To ascertain the cellular source of 8-IsoP and PGE2 in EBC, invasive studies, such as bronchial biopsies, are required. In view of the fact that 8-IsoP is produced by the free radical-induced peroxidation of arachidonic acid on plasma membrane phospholipids, all the cells in the airways may release this eicosanoid.

#### 2.1.4. Chronic and Acute Exposures

Chronic exposures are associated with airway acidity, whereas acute exposures are more closely associated with oxidative stress. Thus, the collection of EBC may contribute to predicting the pathological state of the airways of workers exposed to acute and chronic factors. Grain workers report adverse respiratory symptoms as a result of exposure to grain dust and endotoxins. Studies have shown that biomarkers in exhaled breath condensate (EBC) vary with the severity of airway inflammation [26].

#### 2.1.5. Chronic Obstructive Pulmonary Disease—COPD

8-IsoPs levels were significantly elevated in the EBC of patients with *chronic obstructive pulmonary disease COPD* (mean 18.1 ± 2.0 *vs.* 5.6 ± 0.7 pg/mL), irrespective of lung function impairment. Therefore, EBC 8-isoPGF_2α_ levels may reflect the extension of lung emphysema in COPD patients [46].

#### 2.1.6. Idiopathic Pulmonary Fibrosis

8-IsoPGF_2α_ has been observed to increase in the EBC of patients with *idiopathic pulmonary fibrosis* [15].

#### 2.1.7. Oxidative Stress in Smokers

The collection of EBC could be useful in the assessment of airway oxidative stress in smokers. However, it was found that levels of 8-isoPGF_2α_ and hydrogen peroxide cannot be reproducibly assessed in exhaled breath condensate from healthy smokers because of their low concentration and/or the lack of sensitivity of the available assays. It was not possible to calculate the within-subject variation in a reliable manner since only three of the 12 smokers exhibited detectable 8-isoPGF_2α_ concentrations (mean 4.6 pg/mL; range 3.9–7.7 pg/mL) [47].

#### 2.1.8. Pneumoconioses

Increased 8-isoPGF_2α_ in the EBC was found in patients with pneumoconioses [48].

#### 2.1.9. Sarcoidosis

Elevated concentrations of 8-isoPGF_2α_ have been reported in BAL fluid and exhaled breath condensate (EBC) in sarcoidosis. Higher concentrations of 8-isoPGF_2α_ in EBC of patients with sarcoidosis were noted, whereas a significant decrease was observed only in patients who received treatment, but not in those with spontaneous remissions. Low initial 8-isoPGF_2α_ is a positive prognostic factor. A decrease of 8-isoPGF_2α_ in treated patients reflects a non-specific effect of treatment and is not related to a mere regression of the disease [49].

#### 2.1.10. Silicosis

Silicosis is a chronic occupational disease caused by the inhalation of crystalline silica particles for prolonged periods, which produces inflammation and tissue destruction followed by remodeling of the extracellular matrix. 8-isoPGF_2α_ has been seen to increase in bronchoalveolar lavage in subjects with interstitial lung diseases, such as cryptogenic fibrosing alveolitis, and recently in EBC of such patients [15,35].

In silicosis, increased levels of 8-isoPGF_2α_ together with leukotriene D4 were found in exhaled breath condensate (73.6 ± 9.9 pg/mL) [50].

#### 2.1.11. Systemic Sclerosis

Systemic sclerosis is a systemic connective tissue disease. Increased level of 8-isoprostane in EBC from patients with systemic sclerosis reflect the inflammatory pattern involving oxidative stress [51].

### 2.2. Standardization of EBC Measurement

Guidelines were published by the American Thoracic Society (ATS) for EBC Measurement in 2005 [52], but the lack of standardization of EBC analysis is currently the principal limitation of this technique making it difficult to compare data obtained in different laboratories. Reference analytical techniques are required to provide definitive evidence for the presence of several biomolecules in EBC and an accurate assessment of their concentrations in this biological fluid. Moreover, several methodological issues need to be addressed before this technique can be considered in the clinical management of patients.

## 3. Composition of EBC Samples

**Figure 6.**
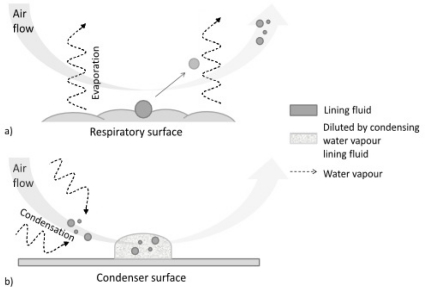
Formation of an exhaled breath sample. (**a**) Respiratory droplets are released from the surfaces of the airways/air spaces. Much greater quantities of water are released as vapor (dotted arrows); (**b**) when the respiratory droplets reach the condenser, they become diluted by large volumes of water vapor that are deposited as large droplets on the walls of the condenser.

Exhaled breath condensate is considered now as a promising source of lung disease biomarkers. It is quite a simple matrix in which biomarkers may be identified, in a way equivalent to blood, sweat, tears, urine and saliva. Figure 6 illustrates the formation of EBC. Exhaled air consists basically of water vapor and substances that reflect the functional status of the lung and other tissues.

EBC usually consists of more than 99.9% of distilled water that condenses from the gas phase out of the nearly water-saturated exhalate (substantially diluting the aerosolized airway lining fluid). This phase contains:

- droplets of variable size (during normal tidal breathing, levels of aerosol particles range between 0.1 and 4 particles/cm^3^, the mean particle diameter is <0.3 μm [53–55]), aerosolized from the airway lining fluid—such particles, presumably reflecting the fluid itself, emanate from the mouth or endotracheal tube in exhaled air [56]; they serve as the only explanation for the presence of clearly non-volatile constituents in EBC such as sodium ions [57].
- water-*soluble* volatiles compounds (exhaled and absorbed in the condensing breath) and non-volatile constituents (compounds mostly derived from the airway lining fluid particles).

There are several mechanisms by which volatile and nonvolatile substances pass from the lungs to the EBC, but first and foremost it is the turbulence developing as air passes that generates sufficient energy for molecules to break away from the airway walls.

Then, the concentration of some of the most frequently analyzed substances in EBC (like hydrogen peroxide) depends on the airflow generated during the procedure, while that of other components (8-IsoP and ethanol) is independent of airflow.

Another mechanism affecting the collection of EBC is the Venturi effect, produced with the opening and closing of the bronchi and alveoli, although little is known of its implications [58].

There is currently no standard invasive or non-invasive method of determining absolute concentrations of airway lining fluid non-volatile constituents with which EBC can be readily compared. However invasively collecting samples from healthy lungs additionally limits knowledge of normal airway fluid components, and normal variability. Invasiveness or discomfort of collection drastically limits the ability to study airway components. These issues underlie the attractiveness of EBC as a research and clinical tool.

## 4. EBC Sample Collection

All the volatile and nonvolatile substances present in EBC can be analyzed by condensing exhaled air by passing it through a cooling unit. As mentioned already, interest in EBC lies first and foremost in its simplicity of collection in nearly any setting. Entirely non-invasive, it usually takes 5–10 min in adults and up to 15–20 min in children to obtain 1–3 mL of condensate, which is a sufficient sample for multiple biomarkers analysis. Only a few studies have used very a short (3 min) or a prolonged (60 min) collection time. The mean volume of air inhaled over 15 minutes was 119 ± 25 L, (95% confidence interval, 112–125 L). The mean volume of condensate in exhaled air over a period of 15 minutes was 1.8 ± 0.5 mL (95% confidence interval, 1.5–2 mL) and the coefficient of variation was 29%. The direct relation between the volume of EBC collected and the volume of air inhaled for each individual is expressed by the following equation: volume of EBC (mL) = 0.013 × volume of air (L) + 0.255 [59]. Direct comparison of different collection times has only been studied with respect to pH: changes in collection time did not show any effect. No difference could be found in the concentrations of 8-isoPGF_2α_ between studies using 10, 15 or 20 min for EBC sampling [52].

The amount of condensate generated per exhalation varies among individuals. Minute ventilation remains the major determinant of the amount of condensate over time, and it does not have any effects on levels of measured substances [60].

There are several different ways of obtaining EBC sample—they are optimized to collect the mediator of interest. Patients are asked to breathe tidally through the mouthpiece, which is connected to a collecting device cooled to 0 °C. Breathing air through a cooling system results in condensation, thereby rendering collection of exhaled breath possible in liquid form. Condensation of exhaled breath can be achieved simply by cooling the tubing through which the patient is exhaling.

The condensing chambers used to date have been made from glass, polystyrene or polypropylene. The composition of the EBC may be altered by the adhesive properties of the material from which the container is made. When analysis of bioactive lipids is planned, polypropylene containers should be considered for collection to avoid the problem of adsorption that can occur with polystyrene containers. There is still no knowledge about whether different surfaces or coating materials interact with different EBC components. In one particular study, glass, Teflon, silicone, aluminum and polypropylene were tested for the recovery of 8-IsoP in EBC [61].

Exhaled air is readily cooled with wet ice as well as with dry ice or liquid nitrogen.

The dead space volume in the collection systems is compared to direct sampling of the mouth or nasal passages. In general, the equipment dead space should be kept as low as possible to avoid significant re-breathing of carbon dioxide and also to minimize condensation or deposition of aerosol on the mask’s inner surface.

Successful collection has been reported with a variety of devices of different designs. The most widely used designs include immersion of Teflon-lined tubing in an ice-filled bucket, and a specially designed double-wall glass condenser system [54].

Several appliances are commercially available for the collection of EBC samples. They are briefly characterized in Table 1.

### 4.1. EcoScreen

**Figure 7.**
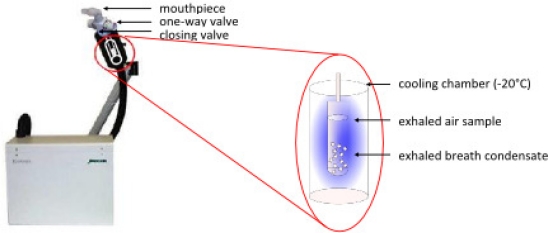
Illustration and diagram of the ECoScreen device for collecting EBC.

The ECoScreen device (Erich Jaeger GmbH, Höchberg, Germany)—an illustration and diagram of which is presented in Figure 7—is an electrical refrigerated system. It has an extendable arm that allows the subject to sit upright on a chair and exhale into a cooled chamber. The condenser maintains a cold temperature (−20 °C) throughout the 5- to 15-minute collection period. As it is the size of a desktop personal computer, however, it is not easily portable. It uses a saliva trap consisting of a hole near the mouthpiece that reportedly keeps the collected fluid amylase-free. Pooling of the condensate requires either sufficient sampling time to overcome surface tension issues or centrifuging of the collection chamber.

The ECoScreen device has important features: It is commercially available, widely used and prevents contamination of EBC with saliva. However, this device may have limitations as exhaled breath condenses on a Teflon coated surface that is repeatedly used. The ECoScreen device is not portable, and the amount of sample is strictly connected with the breathing time. Recently, it was reported that NO_x_ measurements might be confounded by the device and represent (partly) contamination with NO_x_ originating from the device itself [71].

### 4.2. Rtube

**Figure 8.**
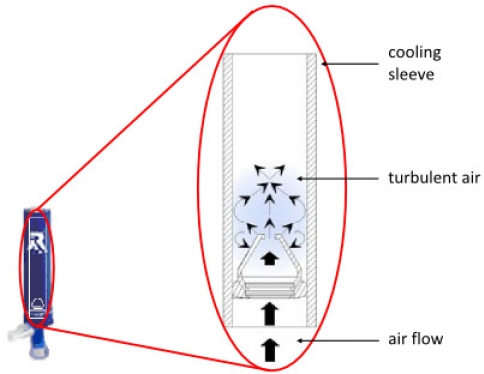
Illustration and diagram of the Rtube device for collecting EBC.

The RTube™ breath condensate collection device shown in Figure 8 is also called a Portable Collection Device and designed for ease of use by the unsupervised patient in the home, workplace, laboratory, hospital or clinic.

This device uses an exhalation valve that also serves as a syringe-style plunger to pool fluid off the condenser walls, allowing for collection times of as little as one minute. Cooling is achieved by placing an aluminum cooling sleeve over the disposable polypropylene condensation chamber, which can also serve as the sample storage container. The temperature of the cooling sleeve can be chosen by the investigator. Single-use design eliminates any possibility of infectious cross-contamination. The device inherently prevents salivary contamination with large a “T” section, in which saliva separates from the exhaled breath, and no evidence of salivary amylase in the condensed fluid has been reported. An attachable microbial filter (0.3 μm) is provided to prevent transmission of infectious particles. Fully self-contained and disposable, this handheld device is therefore clean and ready for use at any time. No cleaning is necessary. As the subject breathes normally into the device, the RTube gathers breath condensate in a transportable cartridge that is easily shipped in the mail for centralized sample analysis. The simplicity and environmental flexibility of this collector offers potential for rapid development of clinical diagnostics utilizing EBC.

Measurements in EBC samples collected by RTube and ECoScreen are repeatable and reproducible in healthy controls, COPD and asthma patients, and subjects with a common cold [72].

However, some authors announced that EBC collection devices influence the efficiency of collection but can also introduce errors in the measurement of total protein levels in EBC.

### 4.3. Other Systems for EBC Collection

A unique home-made system for EBC collection has been presented in the literature recently [73]. Respiratory droplets can be collected by procedures other than condensation on cold surfaces, e.g., filtration, impaction, and electrostatic precipitation on devices at body temperature. After collection the solutes can be washed off with small volumes of water. These alternative techniques may reduce the amount of unwanted water vapor and volatile buffers, such as ammonium, but may not be suitable for thermally labile or volatile mediators [74].

**Figure 9.**
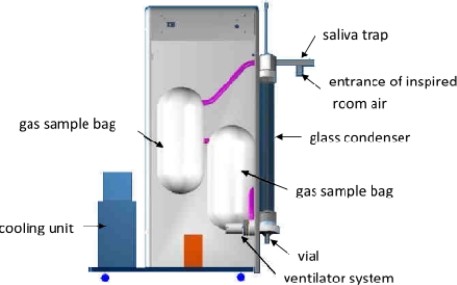
Schematic representation of the glass closed condenser. Inclined glass condenser with a moveable plunger; swan-neck tubing (saliva trap) and two-way non-rebreathing valve, connected to a face mask with separated nose and mouth cavity, entrance of inspired room air; cooling unit; sample vial to collect EBC; ventilator system for recirculation of non-condensed exhaled breath; heated (at 37 °C); inert Tedlar™ gas sample bag to collect the residual non-condensed exhaled breath.

The Department of Instrument Development Engineering and Evaluation of the Maastricht University Medical Center has published a new design of an EBC collection system for use in young children [75]. Figure 9 shows a schematic representation of this closed glass condenser. In short, children breathe tidally for 10 minutes through a mask connected to the two-way non-rebreathing valve. EBC is collected using a cooled double-jacketed glass condenser that is connected to the valve via a tube. The two-way valve and tubing serve as a trap to minimize salivary contamination. Circulating ice water cools the condenser to 0 °C. During this procedure small droplets of breath condensate are formed which is collected in a tube; while this is happening, children can watch cartoons. Exhaled breath that does not directly condense in the condenser is collected in an inert bag connected to the condenser. To prevent the exhaled breath from condensing in the bag, it is placed in a heated box (37 °C). When the child finishes the procedure, the exhaled breath that is temporarily collected in the inert bag is conducted through the condenser (recirculation) to increase the amount of condensate. After collection, EBC is rapidly frozen at −80 °C using dry ice, then stored at −80 °C until analysis.

**Figure 10.**
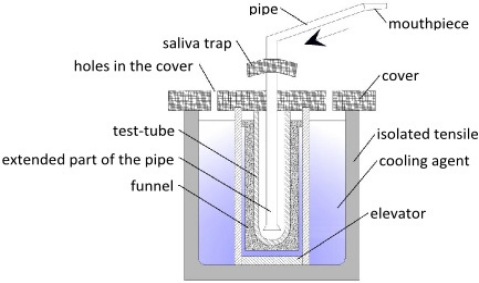
Diagram of the device constructed at the Medical University of Łódź.

A new device has been patented at the Medical University of Łódź, Poland (see Figure 10). A tube for sample collection is placed in an isolated tensile filled with cooling liquid (dry ice, liquefied air, ice or water). If necessary, a bigger test-tube can be inserted, depending on the further analysis of the sample collected. Through the hole in the cover, a pipe with mouthpiece is placed inside the device. The lower part of the pipe is extended, which prevents the condensate from escaping with the air flow. The pipe also has a saliva catcher to prevent contamination of the sample. Holes in the cover enable sublimation or evaporation of cooling agent. An elevator maintains the test-tube in maximum contact with the cooling agent. Water vapor, particles and bronchial secretions condense and freeze on the walls of test-tube. Dried air (due to the pressure obtained from the breath of the test objects) leaves the device through the holes in the cover or is passed to the respiratory system. However, after every use, this device must be dismantled and cleaned. This device for collecting exhaled breath condensate enables samples to be collected even from unconscious patients.

There is currently no standard invasive or non-invasive method of determining absolute concentrations of the non-volatile, constituents of the airway lining with which EBC can be readily compared. However, the invasive collection of samples from healthy lungs additionally limits knowledge of normal airway fluid components, and normal variability. Invasiveness or discomfort of collection drastically limits ability to study airway components. These issues underlie the attractiveness of EBC as a research and clinical tool. There is a clear and urgent need for standardized methods of EBC collection and storage for certain individual biomarkers.

## 5. Salivary Contamination of EBC

In oral EBC collection, depending in part on the EBC collection equipment, gross or microscopic salivary contamination of EBC may and sometimes does occur [76]. Measures of salivary amylase are often used to test for the presence of salivary contamination. Most investigators find that no amylase is identified in the great majority of samples. Higher sensitivity assays tend to report the presence of measurable amylase in a subset of samples [76]. Saliva is the source of less than 10% of respiratory droplets [77]. The ratios among various non-volatile compounds in EBC has been found to be substantially different from the ratio of compounds in saliva, suggesting a dominant (but not entire) lower airway source of EBC constituents [77]. If care is taken to exclude saliva from EBC samples, amylase can be detected in only a small portion of samples with levels approximately 10000 times lower than those in saliva [78]. In addition, amylase measurements are not specific to salivary amylase, and amylase can also be found in the lungs, so positive results of the test do not necessarily mean salivary contamination. Some solutes measured in EBC such as eicosanoids have been examined as markers of salivary contamination as well [79–80]. Even when collected free of saliva contamination the majority of proteins present in EBC were also found in saliva, suggesting that these proteins are present in both compartments, e.g., saliva and secretions of the lower airspaces. The quantification and identification of specific proteins in the various compartments is warranted in future studies to determine the practical value of EBC.

## 6. Storage of EBC Samples

Because on-line measurements are not available for most biomarkers present in EBC with the exception of a few assays including those for pH and H_2_O_2_, EBC samples are usually stored before final measurements. EBC samples should be immediately frozen after collection and stored at −70 °C until biomarker determination is performed. Another case to consider is the number of target compounds. Samples of EBC should be divided into parts and stored separately. At present, very little is known about the reproducibility and stability of biomarkers during long term storage [81]. However, it is well known that multiple frosting-defrosting cycles could destroy some biomarkers like prostaglandins [52]. Even when stored under these conditions, some analytes are not stable after long-term storage. For example, the concentration of H_2_O_2_ is known to decrease causing detectable change in its level after a few weeks at −20 °C [82,83]. Cysteinyl-leukotrienes (cys-LTs) are also unstable compounds in most biological fluids, although data on their stability in EBC is not well documented. Isoprostanes stability has been studied by several authors [25,46]. On the other hand pH has been reported to be stable up to two years of storage [82].

Also, other types of samples can be successfully stored over long periods of time. Samples of blood plasma immediately frozen in liquid nitrogen and stored at −80 °C exhibit no auto-oxidation for up to eight months. Similar precalculations have to be considered when handling the solid tissue samples [2].

## 7. Sample Preparation

Since the majority of the sample consists of condensed water vapor, most biomarkers exist in EBC at very low concentrations. Sample preconcentration is necessary to improve the limits of detection (LODs) of any analytical method. Isoprostanes can be preconcentrated from EBC samples by either lyophilisation or adsorption on sorbent traps (SPE).

### 7.1. Lyophilization

Lyophilization as an exceptional technique for concentrating non-volatile (or semi-volatile) substances dissolved in water. Since this technique is extremely suitable for heat-labile substances, it is fairly often used as the preconcentration stage for 8-isoprostane contained in EBC. During freeze drying of the EBC sample, no temperature stress on the labile markers of oxidative stress occurs, which could lead to the preference of lyophilization over other potential pre-treatment techniques. Lyophilization is based on dehydrating a delicate sample that would otherwise be damaged by the drying process. By regulating the temperature and pressure of the sample, freeze-drying brings the sample system around the triple point of a typical phase diagram, thereby avoiding the liquid–gas transition seen in ordinary drying. After lyophilization is complete, all free water is removed and the solute remains. This solute can then be reconstituted in a solution of choice. However, in contrast to this temperature advantage, there is also no separation capability from the other non-volatile substances present in EBC, which are concentrated as well. This could result in a deteriorated ionization during the mass spectrometric measurement or possibly elevated noise even in preceding liquid chromatography step. This is apparently, caused by the presence of a high amount of substances including salts. This fact could be negatively reflected in higher values of LOD and LOQ as well as poorer method accuracy in low (picogram) concentrations compared to other potential pre-treatment methods. On the other hand, the EBC matrix is composed mainly of water, whereas the content of non-volatile substances and salts is relatively low compared to other body fluids (urine, blood plasma, cerebrospinal fluid).

So far, lyophilization has been used by many research groups evaluating EBC biomarkers [84–87]. A 1mL EBC sample lyophilized and resuspended into 50 μL of mobile phase used for further analysis gives a 20-fold concentration. Reconstituted volumes can be applied to multiple immunoassays [84] or HPLC determination [85].

### 7.2. Solid Phase Extraction

Solid phase extraction (SPE) is a technique designed for rapid, selective sample preparation and purification prior to chromatographic analysis. Using liquid chromatography principles to control selectivity, SPE provides the sample clean-up, recovery, and concentration necessary for accurate quantitative analysis. Over the last twenty years, SPE has become the most powerful technique available for rapid and selective sample preparation and preconcentration. The concentration of analytes for increased sensitivity is the main aim of applying SPE to EBC. General guidelines for choosing the appropriate SPE tube size, bed weight and sorbent type are available. Nevertheless, optimal method parameters should be determined during method optimization. Providing the right adsorbent is chosen, the extraction process can be very efficient, e.g., >99%. Although, solid phase extraction is an extremely efficient method for isolating and concentrating solutes from relatively large volumes of liquid, this technique can be very effective, even when the solutes are present at extremely dilute concentrations (e.g., ppb) and in small sample volumes. Generally, 3 mL SPE tubes are the most common size. However, smaller tube volumes (1 mL), which contain smaller bed weights, allow for reduced elution volumes, which can be beneficial for sensitive analyses, as in the case of biomarker determination in EBC samples. Materials extracted by SPE can be used for subsequent chromatographic separation, spectroscopic examination or biological assessment. For isoprostane extraction and concentration from EBC, which contains large amounts of water, the reversed phase mode is usually applicable.

In order to obtain good results using SPE, there are basic steps to perform:

- *Sample Pretreatment* for interferent-laden samples (e.g., biological fluids)—dilute samples 1:1 with buffer. pH manipulation may be important in the case of ionizable compounds. A compound’s ionization state can drastically change its retention and elution characteristics on a given SPE sorbent. When an analyte is in its neutral form, it becomes more hydrophobic and retention strengthens under reversed-phase conditions. Adjusting the sample pH to 2 pH units above or below the compound’s p*Ka* (depending on the functional group) will effectively neutralize the compound. Non-polar solvents (including methanol and isopropanol) prevent interaction between the compound and sorbent functional groups. To avoid clogging, it may be necessary to centrifuge, or pre-filter the sample prior to introducing it to the SPE phase.
- *Conditioning/Equilibration* wets or activates the bonded phases to ensure consistent interaction between the analyte and the sorbent functional groups. Reversed-phase sorbents are often conditioned with 1–2 tube volumes of a water-miscible solvent such as methanol or acetonitrile. Equilibration introduces a solution similar to the sample load in terms of solvent strength and pH in order to maximize retention. 1–2 tube volumes of buffer (used in sample pre-treatment) or water are good choices for reversed-phase equilibration.
- *Sample Loading* must be done at a consistent and reduced flow rate of ∼1–2 drops per second to ensure optimal retention.
- *Washing* of sample interferences that are often co-retained with target compounds during sample loading. A washing step is necessary to elute interferences without prematurely eluting target compounds. 5–20% methanol in water or sample pre-treatment buffer is typical for washing solvents.
- *Elution* can be performed with an organic solvent or solvent combination of sufficient non-polar character in order to prevent hydrophobic interactions between the analyte and sorbent functional groups. Example elution solvents are 1–2 volumes of methanol or acetonitrile. pH manipulation during elution can often improve recovery in the case of ionizable compounds. In their ionic form, basic and acidic compounds become more polar, attenuating reversed-phase interaction, so weaker elution solvents and/or reduced elution volumes can be used.
- *Eluate Post-treatment* is often necessary in, for example evaporation to dryness and reconstitution of SPE eluate in the mobile phase prior to LC analysis. GC analysis often requires further SPE eluate concentration, possible matrix exchange with a more volatile solvent and/or derivatization.

Several authors have reported the application of SPE for IsoPs preconcentration from EBC samples. Generally, two types of SPE sorbents are applied: RP-18 sorbents [88–90] and immunoaffinity sorbents [14,17,89]. In table 2 are presented procedures most common used for EBC sample preparation.

## 8. Determination of Isoprostanes in EBC Samples

A variety of analytical techniques are involved in the final step, which enables identification and quantitative analysis of the compounds present in exhaled breath condensates, from metabolic end products to proteins, to a diversity of cytokines and chemokines. Most of the mediators found in EBC samples are in the lower range of detection of the techniques currently available, where the intra- and inter-assay variability of methods is large. An option to overcome this problem is sample preconcentration by SPE, lyophilization and vacuum evaporation, as discussed earlier. Volatile, semi-volatile and unstable substances are very likely to be lost during freeze-drying.

**Figure 11.**
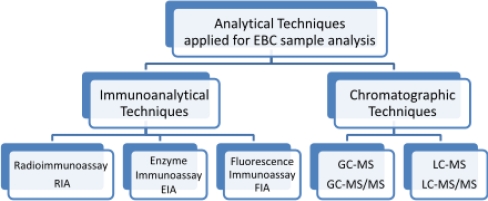
The analytical techniques most commonly used to determine isoprostanes in exhaled breath condensates.

Determination of IsoPs in exhaled breath condensates is based on immunoanalytical and chromatographic techniques (Figure 11). Radioimmunoassay (RIA) is one of the most efficient, convenient and accurate techniques for the detection of small amounts of analytes in fluids, because radioactivity is fairly free of matrix interferences and can be measured in small amounts. RIA is cost-effective, specific, sensitive and does not require sample preparation. Because dilution of respiratory droplets by water vapor may explain part of the variation in concentrations of non-volatile compounds in EBC, results can be presented as a change in the ratio the target analyte concentration to the concentration of another eicosanoids [91]. RIA or enzyme immunoassay (EIA) techniques offer ease of performance, the possibility of automation and low cost of analysis. In most published studies, IsoPs have been measured using commercially available enzyme immunoassays [25,46,63,92–95]. However, the available ELISA kits were originally validated for matrices other than EBC, such as urine, plasma or serum. Quantification by ELISA-kits could be influenced by the matrix in which the analytes are dissolved. In view of the extremely high dilution of the epithelial lining fluid in EBC (and the resulting low concentrations), EBC can be considered to contain only little matrix compared to other highly concentrated and protein-rich matrices such as plasma or urine. EBC is characterized by low concentrations of proteins and amino acids, so that matrix influences should be minimal, both for ELISA and more specific analytical techniques, e.g., gas chromatography mass spectrometry (GC-MS) or liquid chromatography mass spectrometry (LC-MS), suggesting good comparability of both detection techniques. A comprehensive comparison of two unrelated analytical techniques was performed in order to quantify the three most frequently analyzed eicosanoids in EBC [96]. The results obtained in this study show that ELISA overestimates the eicosanoids in EBC. On the other hand, a method developed for determining of 8-*iso*-PGF_2α_ in EBC from asthmatics, shows acceptable reproducibility of EIA compared to GC/NICI-MS, even if the latter method had higher accuracy [45].

Therefore, quantification by immunoassays has to be validated by GC-MS/MS or LC-MS to provide definitive evidence for accurate quantitative biomarkers results in EBC.

### 8.1. Gas Chromatography with Mass Spectrometry and Tandem Mass Spectrometry Detection

The presence of 8-*iso*-PGF_2α_ was confirmed for the first time in EBC using stable isotope dilution method in conjunction with GC-MS [97]. Since that time, analysis of EBC has hardly ever been done by GC-MS and GC-MS/MS due to derivatization of analytes. It is more often applied to samples like urine, plasma or tissues, where the complicated sample preparation usually requires at least one extraction step and one purification step. Derivatizing the carboxylic acid, common to all eicosanoids, to the pentafluorobenzyl ester is the key to its sensitivity. The reagents most commonly used for derivatization are *N*,*N*-Diisopropylethylamine (DIPEA) with Pentafluorobenzyl bromide (PFBBr) [98] and Bis(Trimethylsilyl)-Trifluoroacetamide (BSTFA) with Dimethylformamide (DMF) [20]. GC-MS and GC-MS/MS techniques are sensitive to quantitate a single F2-isoprostane isomer in a 1 mL sample but not specific enough to separate F2-isoprostane isomers from each other. A method was found where prostaglandins, 8-isoPGF_2α_ and 3-nitrotyrosine were detected in EBC from asthmatics and healthy non-smokers. In all asthmatics and controls, prostaglandins, 8-isoprostane, and 3-nitrotyrosine were not detectable by GC-MS or LC-MS analysis [99].

### 8.2. Liquid Chromatography with Mass Spectrometry and Tandem Mass Spectrometry Detection

In the past decade, LC has become the practical alternative to gas chromatography. This is because no derivatization is needed for semi-volatile and non-volatile compounds, which are thermolabile. The two main ionization modes used in current commercial instruments are electrospray ionization and atmospheric pressure chemical ionization (APCI). Different acquisition modes are available in modern LC-MS/MS instruments. During qualitative determination, the full-scan mode is useful where a range of *m*/*z* values encompassing the analytes of interest is constantly monitored. Based on these results, pseudomolecular and fragmentation ions can be obtained, so molecular mass and fragments may help to identify analytes. On the other hand, multiple reaction monitoring (MRM mode) is in common use. In this mode, a specific set of precursor ions is selected and their transition from the selected precursor ion to a specific product ion is monitored. This reduces chemical noise leading to higher mass spectral sensitivity and selectivity, which is used for quantitative determination.

Weakly acidic isoprostane isomers (p*Ka* ∼ 5) can be deprotonated to form (M-H) precursor ions in the electrospray ionization source and detected in the negative ion mode. MS/MS can be used to fragment the precursor ions and form specific product ions. The multiple reaction-monitoring (MRM) mode is used for its extremely high degree of selectivity and the stable isotope dilution assay for its high precision of quantification. The scan monitoring reaction used for analyses were 353→193 for 8-*iso*-PGF_2α_ and 357→197 for [3,3’,4,4’ ^2^H_4_] 8-*iso-*PGF_2α_.

The application of liquid chromatography/mass spectrometry (LC-MS/MS) has a few advantages: Unlike to GC-MS, it does not require the two-step derivatization procedure, which means improved recovery, a shorter sample pre-treatment time, and the absence of incomplete derivatization by-products. Examples of chromatographic parameters for determining IsoPs in biological samples are presented in Table 3.

Tandem mass spectrometry coupled with LC offers the high sensitivity, specificity, and accuracy that are required for biomarkers determination in EBC.

## 9. Trends and Future Directions

EBC analysis is expanding on its promise of expanding the number of mediators that can be measured to provide clinically useful methods by which to measure and monitor therapy, biochemical processes, alterations in air flow (characteristics) and inflammatory lung diseases [58]. The main aspects of future research will include investigation into the mechanism of EBC formation and studies of its relationship with airway lining fluid; the correlation of lung function and symptoms with markers occurring in EBC; and reproducibility of measurements [100]. To improve the routine use of EBC, numerous issues must be discussed, for example, matching appropriate, sensitive and specific assays to detect biomarkers, and the reference values for markers found in EBC to assist in diagnosis and treatment [101].

Proteomics, which applies high resolution gel electrophoresis or MS to detect multiple proteins in biological samples, may also be a useful approach to analyse proteins in EBC. The molecules detected, e.g., ribonucleic acid (RNA), deoxyribonucleic acid (DNA) and microorganisms, contribute to development of research in fields like microbiology and gene delivery. This may reveal disease-specific patterns and may lead to the identification of novel proteins for the detection of disease and the identification of new therapeutic targets. However, there are several technical problems that need to be overcome before this becomes a useful approach [54].

Metabonomics is a recent technique that may be particularly applicable to EBC analysis. It involves the detection of hundreds of thousands of metabolites in a biological fluid usually using high resolution nuclear magnetic resonance spectrometry or liquid chromatography/MS. Although less sensitive than ELISA and mass spectrometry, NMR requires minimal sample preparation, with a rapid acquisition time (10–15 min), and has a high degree of sensitivity (less than or equivalent to μmol/L levels). Furthermore, it is nondestructive and allows complete detection of observable metabolites (“sample metabolic fingerprint”) at a reasonable cost, screening of lung diseases, following disease progression, predicting responses to treatment and monitoring of response to therapy [102,103].

## 10. Conclusion

The use of exhaled breath condensate is promising because it is a simple, noninvasive technique that can be compared with invasive protocols for assessing lung inflammation like BAL. The low cost of collection related with high reproducibility, will permit real-time, repeated measurements of pulmonary diseases. The determination of the role of EBC in diagnosis and management of individual patients, an issue distinct from large studies of disease mechanisms, awaits further investigation. It could also provide insight into the pathophysiology of inflammatory lung diseases and its potential usefulness for assessing the efficacy of drug therapy.

On the other hand limitations like the lack of a standard method of collection, high levels of interfering substances and low concentrations of biomarkers are the main drawbacks of EBC analysis. Further studies need to be done on the mechanism of EBC formation. In order to introduce breath analysis into clinical practice, analytical procedures have to be considerably simplified. For this purpose, analytical devices have to be portable, be able to detect markers without delay, and be easy to use. These requirements can best be met by a smart combination of sampling preparation and detection techniques.

## Figures and Tables

**Table 1.** Commercially available exhaled breath condensate collection systems.

**EBC Collection System (Manufacturer)**	**Advantages**	**Disadvantages**	**References**
ECoScreen I and ECoScreen II (Viasys, U.S., Europe)	The most commonly applied EBC collection system. More often used in European countries. There is an optional package for determining the total exhaled volume.	Not readily portable. Cleaning between patients may need to be extensive to abide by standard respiratory care practices. No way of controlling condensation temperature (Eco1).	[13,29,44,49,62–63]
Rtube (Respiratory Research, U.S.)	More total EBC collections than other systems. Multiple collections can be performed concurrently. Most commonly applied in North American centers. No cleaning between patients is necessary. Portable. Can be prepared for use in a standard freezer.	Choice and maintenance of set condensing temperature requires an optional cooling unit, otherwise the condensation temperature is chosen by cooling the sleeve preparation temperature and rises during collection.	[64–66]
Anacon (Biostec, Valencia, Spain)	Temperature of collection can be controlled. Designed for use on ventilated patients	Only a few publications are available.	[67,68]
TurboDeccs (ItalChill, Parma, Italy)	Has both non-disposable and disposable portions. Controllable collection temperature. Moderately portable. Readily cleanable because components are disposable.	Very few publications exist. Only one collection at a time is possible.	[69]
Polish Patent	Constant maximum contact between the test-tube and the cooling agent. Possibility of inserting different sizes of test tubes.	Dismantling and cleaning after use is necessary.	[70]

**Table 2.** Examples of sample preparation procedures for EBC samples with different final analysis techniques.

**Analytical Technique**	**Sample Preparation**	**LOD**	**Ref.**
RIA	standard curves obtained using phosphate buffer 0.025 mol/L, pH 7.5, more similar to EBC matrix	10 pg/mL	[91]
	samples stored at −80 °C before analysis	10 pg/mL	[36]
EIA	after sampling no further preparation of the sample is necessary, samples stored at −80 °C before analysis	4–5 g/mL	[25,46,63, 92–95]
	SPE of pooleed sample (35.5 mL), HPLC purification	10 pg/mL	[90]
LC-MS/MS	samples pretreated with 50 μL of immunoaffinity sorbent for 60 min	1 pg/mL	[17]
	after collection sample spiked with 8-isoPGF_2α_-d_4_, stored at −80 °C, lyophilization, redissolved in 50 μL of water/methanol (1:1)	8 pg/mL	[85]
	sample spiked with 8-isoPGF_2α_-d_4_, pretreated with 50 μL of immunoaffinity sorbent for 60min at 20 °C, centrifuged (3000 rpm for 5 min), immunoaffinity sorbent flushed twice with water, elution with methanol, methanol evaporated, redissolved in 50 μL of mobile phase	1 pg/mL	[14]
LC-MS	immunoaffinity separation	1 pg/mL	[16]

**Table 3.** Chromatographic parameters in the analysis of biological samples for isoprostanes’ determination.

**Specimen**	**Determined Analyte**	**Column**	**Eluents**	**Ref.**
EBC	8-*iso*-PGF_2α_	Hypercarb Thermo 100 mm × 2.1 mm × 5 μm	A: acetonitrile B: water (pH 11) isocratic elution A:B (7:3) flow rate 250 μL/min	[14,17]
EBC	8-*iso*-PGF_2α_ *o*-Tyr 8-OHdG	Hypercarb Thermo 100 mm × 2.1 mm × 5 μm	A: aqueous solution of ammonium hydroxide pH 10.5 B: methanol:acetonitrile 60:40 (v/v) + 0.1% of ammonium hydroxide gradient elution	[85]
Urine Plasma	8-*iso*-PGF_2α_	Symmetry C8 150 mm × 3.9 mm × 5 μm	A: 0.1% acetic acid (pH 3) B: acetonitrile isocratic elution A:B (7:3)	[8]
Urine	8-*iso*-PGF_2α_	YMC ODS-AQ 50 mm × 2.0 mm × 3 μm	A: methanol: acetonitrile 5:95(v/v) B: 2 mM ammonium acetate gradient elution flow rate 0.2 mL/min	[7]
Urine	8-*iso*-PGF_2α_	Phenomenex Gemini C 18 110 Å, 50mm × 2.0 mm × 3 μm	A: 0.1% aqueous solution of ammonium hydroxide B: 0.1% ammonium hydroxide in acetonitrile gradient elution	[9]
Plasma	8-*iso*-15(R)PGF_2α_ 11β-PGF_2α_ 15(R)-PGF_2α_ PGF_2α_	Synergi Hydro-RP 250 mm × 2.0mm	A: water + 0.01% acetic acid B: methanol gradient elution	[10]
Urine	8-*iso*-PGF_2α_ 8-*iso*-15(R)-PGF_2α_ 8-*iso*-PGF_2ß_ PGF_2α_	Hypercarb, 5 μm, 1 mm × 150 mm, porous graphitic carbon column	A: water + 0.5% aqueous solution of ammonium hydroxide (pH 9.5) B: acetonitrile:methanol 40:60(v/v) + 0.5% aqueous solution of ammonium hydroxide flow rate 50 μL/min gradient elution	[6]
